# Advances in Multi-Functional Ligands and the Need for Metal-Related Pharmacology for the Management of Alzheimer Disease

**DOI:** 10.3389/fphar.2018.01247

**Published:** 2018-11-15

**Authors:** Abha Sharma, Vidhu Pachauri, S. J. S. Flora

**Affiliations:** Department of Pharmacology and Toxicology and Department of Medicinal Chemistry, National Institute of Pharmaceutical Education and Research, Raebareli, India

**Keywords:** neurodegeneration, multi-functional ligands, metal chelation, antioxidant, Aβ inhibition

## Abstract

Alzheimer’s disease (AD) is the age linked neurodegenerative disorder with no disease modifying therapy currently available. The available therapy only offers short term symptomatic relief. Several hypotheses have been suggested for the pathogenesis of the disease while the molecules developed as possible therapeutic agent in the last decade, largely failed in the clinical trials. Several factors like tau protein hyperphosphorylation, amyloid-β (Aβ) peptide aggregation, decline in acetyl cholinesterase and oxidative stress might be contributing toward the pathogenesis of AD. Additionally, biometals dyshomeostasis (Iron, Copper, and Zinc) in the brain are also reported to be involved in the pathogenesis of AD. Thus, targeting these metal ions may be an effective strategy for the development of a drug to treat AD. Chelation therapy is currently employed for the metal intoxication but we lack a safe and effective chelating agents with additional biological properties for their possible use as multi target directed ligands for a complex disease like AD. Chelating agents possess the ability to disaggregate Aβ aggregation, dissolve amyloid plaques, and delay the cognitive impairment. Thus there is an urgent need to develop disease modifying therapeutic molecules with multiple beneficial features like targeting more than one factor responsible of the disease. These molecules, as disease modifying therapeutic agents for AD, should possess the potential to inhibit Aβ-metal interactions, the formation of toxic Aβ aggregates; and the capacity to reinstate metal homeostasis.

## Introduction

Alzheimer’s disease (AD) is a complex neurodegenerative disease characterized by gradual loss of neurons which may lead to decline in learning ability, loss of memory, and other cognitive functions. AD is the most common cause of dementia in elderly people above the age of 65 (Goedert and Spillantini, 2006; Finder, 2010). Nearly 44 million people worldwide suffer with AD and number are likely to reach 76 million in 2030, and 135 million in 2050 (Prince et al., 2013). Numerous studies available in literature to understand the pathogenesis of AD, suggest that multiple factors such as low levels of acetylcholine, mitochondrial dysfunction, β-amyloid (Aβ) deposits, tau-protein aggregation, oxidative stress, inflammation, and dyshomeostasis of biometals might be playing a crucial role in the pathogenesis of AD (Zhang, 2005; Mandel et al., 2007; Henry et al., 2010; DeToma et al., 2012; Kotler et al., 2014; Pfaender and Grabrucker, 2014). AD cannot be detected at the early stage of the development and it is difficult to identify biomarkers that predict disease before behavioral symptoms and memory loss. Studies primarily focus on the detection of Aβ and tau protein as AD biomarkers (Kim et al., 2018). Additionally, numerous genomics, proteomics, and metabolomic studies have identified biomarkers that are helpful in prediction of disease development from mild cognitive impairment to AD. Other than such biomarkers, monitoring of metal ion concentration in the blood/plasma can also be employed for the diagnosis of AD (Hajipour et al., 2017). The oldest one however, is the cholinergic hypothesis that states “decrements in the functional integrity of cholinergic neurons underlie diminished cognitive function in aged subjects and those with AD” (Terry and Buccafusco, 2003). Changes in the central cholinergic system have been well-correlated with the cognitive and non-cognitive symptoms in AD (Akasofu et al., 2008; Dumas and Newhouse, 2011; Tai et al., 2012). The oldest/current therapies include cholinesterase inhibitors (i.e., tacrine, donepezil, rivastigmine, and galantamine), and partial *N*-methyl-D-aspartate (NMDA) antagonists (Massoud and Léger, 2011). These drugs only provide modest cognitive, behavioral and functional symptomatic improvement without addressing the etiopathologic progression of the disease. Thus, in absence of disease altering agents, a novel approach to drug discovery/or therapeutic strategy for AD cure is awaited.

The biggest risk factor for AD is the age that directly correlates with metal dyshomeostasis. Several clinical studies have reported that the concentrations of metal ions are altered in AD patients compared to their age corrected controls (Opazo et al., 2002; Dong et al., 2003; Zatta et al., 2009; Kenche and Barnham, 2011; Loef and Walach, 2012; He et al., 2013). Iron, zinc, and copper levels appear to be dysregulated in AD (Salvador et al., 2010) and imbalance in their levels have been linked to cognitive loss and neurodegeneration (Sastre et al., 2015). It may be noted that enzymes such as monoaminooxydase B, important for the degradation of certain neurotransmitters, is modulated by metals like aluminum (Zatta et al., 1999). This is the basis for the “Metals Hypothesis of AD,” which states that maintenance of metal homeostasis is crucial for neuronal function (Bush, 2013; Sastre et al., 2015). Further, amyloid plaque that defines AD is known to possess high levels of zinc (1055 mM), iron (940 mM), and copper (390 mM) (Lovell et al., 1998). Thus, relevant evidences connecting biometal dyshomeostasis with AD pathogenesis have been described in the present review before addressing them as novel therapeutic targets. Agents that can restore metal dyshomeostasis may be efficient to alter disease pathogenesis. However, recent research in the discipline highlights the complex multi-factorial pathology of AD, formulating consensus for the need to adapt multiple target therapy. A decade old concept of multi target directed ligands (MTDLs) that described compounds effective in treating complex diseases because of their ability to interact with the multiple targets thought to be responsible for the disease pathogenesis have been explored in AD (Talevi, 2015). In contradiction to ‘one molecule one target’ theory, MTDL or multi-functional ligands target more than one bio-molecule with moderate affinity thus aiming to modulate multiple pathways possibly at specific organ or tissue level. Having said that, the classical chelation therapy may not be the simple answer to AD, rather existing molecules and/or newer agents will have to be accessed for restoring metal deregulation (Barnham and Bush, 2014). Interestingly, no drug has 100% affinity or precision to its target and so are chelating agents that invariably alter essential metal distribution. Thus, revisiting these therapeutic agents to design novel multi-functional ligands by adding functional groups targeting relevant other AD pathways is required.

## Biomarker for Alzheimer’s Disease Diagnosis

Early diagnosis of AD is difficult as initial symptoms of the disease overlap with the other types of neurodegenerative disorder such as vascular dementia, frontotemporal lobe, and Lewy body dementia. AD is diagnosed by the evaluation of illness history, pattern of cognitive deficits, memory tests, and laboratory tests such as blood tests, neuroimaging of the brain and evaluation of biomarkers. By evaluation of pathological changes in certain biomolecules in AD might aid in diagnosis and prognosis of different dementias. However, an ideal biomarker should be capable of distinguishing AD from other types of dementia (Humpel, 2011). The Aβ deposition in the brain can be detected before the onset of clinical manifestation. AD is diagnosed by measuring concentration of Aβ_1–42_, phospho-tau-181 and total tau in cerebrospinal fluid (CSF). The concentration of Aβ_1–42_ in CSF can be measured by techniques such as enzyme-linked immunosorbent assay (ELISA), mass spectrometry and microarrays (Oláh et al., 2015; Kuhlmann et al., 2017). A significant drop in CSF Aβ_1–42_ levels in AD patients in comparison to control subjects is suggestive of impaired clearance as well also accumulation of Aβ inside AD brains. Accumulation of Aβ inside brain can be visualized and quantified using PET imaging techniques. The Positron emission tomography (PET) imaging tracers can be useful in monitoring Aβ aggregation in the brain (Marcus et al., 2014; Blennow et al., 2015). The ^11^C and ^18^F labeled chemical probes like thioflavin-T known as Pittsburgh Compound-B, ^18^F-flutemetamol, ^18^F-florbetapir, and ^18^F-florbetaben for Aβ PET imaging have been developed. ^18^F amyloid tracers is having long half life compared to ^11^C labeled tracers therefore former amyloid tracers are approved for clinical use (Morbelli and Bauckneht, 2018). A novel technique “gradient echo plural contrast imaging” was investigated for the *in vivo* determination of Aβ accumulation at the early stages of AD (Zhao et al., 2017). On the other hand, it is quite challenging to develop robust blood based biomarkers for Aβ pathology. Correlation of plasma Aβ proteins with cerebral β-amyloidosis have been found clinically significant in AD diagnosis as shown by some reports (Kaneko et al., 2014; Janelidze et al., 2016; Ovod et al., 2017; Nakamura et al., 2018). Several other plasma proteins like, immunoglobulin M, α2-macroglobulin, chemokine ligand 13, pancreatic polypeptide Y, vascular cell adhesion protein 1, apolipoprotein A, and interleukin 17 were also study for their any correlation in determination of Aβ aggregation in brain. However, results of the studies are promising but to approve as clinical biomarkers for AD diagnosis needs further validation (Voyle et al., 2015; Burnham et al., 2016; Westwood et al., 2016). Other pathological parameters are also helpful in AD diagnosis such as measure the concentration of tau protein in CSF, blood, and visualize neurofibrillary tangles mainly composed of hyperphosphorylated tau in the brain by using PET tracers (Chhatwal et al., 2016; Okamura et al., 2016; Olsson et al., 2016; Winston et al., 2016; Saint-Aubert et al., 2017). Elevation of CSF- phosphorylated tau levels in AD patients in comparison to normal subjects is of particular interest. Similarly, accumulation of tau pathology in brain can be visualized by several tau PET tracers which are in various phases of clinical development. Importantly, there is no reliable blood biomarker for tau protein detection. Tremendous progress has been done to identify and validate blood based proteins that can use as biomarker of AD diagnosis (Shi et al., 2018). However, studies have reported an ultrasensitive techniques like immunomagnetic reduction technology, single molecule array technology and measure phosphorylated-tau level in neuronally derived blood exosomes (Lue et al., 2017). Efforts have been made to establish ratio of Aβ42/tau in CSF as biomarker for clinical application in AD diagnosis (Ritchie et al., 2017). Apart from above mentioned biomarkers, many other pathological changes involved in AD such as oxidative stress, mitochondria dysfunction, axonal degeneration (neurofilament light and total-tau), synaptic degeneration (neurogranin), glial activation, and protein dysfunction can be measured and developed as clinical biomarker (Mantzavinos and Alexiou, 2017; Lashley et al., 2018).

## Therapeutic Drug Targets of Alzheimer’s Disease

The concept of multi-functional drugs has been widely accepted owing to the therapeutic failures of conventional therapy in complicated diseases like AD. The multiple patho-etiological pathway of AD has conventionally targeted its neurological hallmarks in isolation. These hallmarks with their molecular targets have been tabulated below (Table 1) for us to later appreciate aiming for multiple targets simultaneously (Smith et al., 2012; Heckman et al., 2015; Hoang et al., 2017; Hung and Fu, 2017; Krajnak and Dahl, 2018).

**Table 1:** Hallmarks and drug targets of AD.

#	Neuropathological hallmark/pathways involved in pathogenesis	Target	Molecules to be developed as
1	Acetylcholine decreases	AChE	Inhibitor
		5-HT_6_ receptor	Antagonist
		Histamine H_3_	
		α7 Nicotinic acetylcholine receptor	Agonist
		(α7nAChR)	
2	Formation of amyloid plaques	β-Secretase (BACE 1)	Inhibitor
		γ-secretase	Inhibitor
		Glutamyl cyclase	Inhibitor
3	Hyperphosphorylation of tau protein	Glycogen synthase kinase 3β	Inhibitor
4	Increases of glutamate	NMDA blockers or glutamate release	Inhibitor
5	Dyshomeostasis of metals	Homeostasis of copper, zinc, and iron	Chelator
6	Neuroinflammation	Microglial activation	Inhibitor
7	Ca^2+^dyshomeostasis leads to endoplasmic reticulum stress	Sarco/endoplasmic reticulum Ca^2+^-ATPase	Activator
8	Overexpression of DYRK1A	Tyrosine phosphorylation regulated kinase-1A (DYRK1A)	Inhibitor
9	Degradation of cyclic nucleotides	Phosphodiesterase	Inhibitor

## Role of Metals in AD Pathogenesis

Role of metal(s) in patho-physiology of AD has been discussed earlier (Kenche and Barnham, 2011). However, it is only recently that metal hypothesis is considered significant, as conventional therapies failed and research established its causative role. Evidence highlighting biometal imbalance being causative during AD pathogenesis, more than toxic metal exposure persuades that the hypothesis is central patho-etiological pathway. Essential metals are crucial for physiological functions at molecular levels and their tight regulation ensures health at cellular level. Thus, it is agreeable that their dyshomeostasis can disrupt the cellular machinery pushing toward neurodegeneration (Toni et al., 2017). High levels of biometals in AD patient brain have been reported (Lovell et al., 1998). Previous authors have highlighted how in absence of passive transport, imbalance of these metals may be attributed to faulty distribution within brain, owing to deregulation of their transporter proteins (Li Y. et al., 2017).

Iron has been found elevated in the brain of AD patient especially in globus pallidus and putamen, without the increase in serum levels (Tao et al., 2014; Wang D. et al., 2014; Wang Z.-X. et al., 2015; Moon et al., 2016). Iron enters the neuron through importers like tranferrin, divalent metal transporter 1 (DMT1), lactoferrin, melanotransferrin and is exported via ferroportin (fpn). Bio-transporters especially DMT1 and fpn may be primarily deregulated (Xian-hui et al., 2015). Interestingly a recent study has suggested CSF ferritin as predictive biomarker for AD (Ayton et al., 2015a). Iron-induced oxidative damage (Fe^+3^) via hydroxyl radicals produced through Fenton’s reaction causes AD-linked protein injuries (Salkovic-Petrisic et al., 2014). The effect of lead compounds on amyloid-β precursor protein (APP) was studied in human neuroblastoma cell lines (SH-SY5Y). The data revealed that excess intracellular iron (Fe^+2^) binds with APP mRNA and promotes translation of the transcript (Rogers et al., 2016). The effect of zinc on the proteolysis of synthetic ApoE reveal that it affects the proteolysis of ApoE may contribute to the pathogenesis of AD. Study has shown that ApoE can alter zinc concentration in the hippocampus which is crucial to maintain normal function of brain (Liu et al., 2010). Study employing ‘Density functional theory’ suggested that Fe^+2^ binding reduced the helix structure and increases the β sheet content in the peptide which promotes the aggregation by enhancing the peptide-peptide interaction. Further, Fe^+2^ promotes tau phosphorylation via activation of cyclic dependent kinase (CDK5) and glycogen synthase kinase 3β (GSK3β) (Lovell et al., 2004). Iron chelator desferrioxamine, exhibited the ability to reduce the iron-induced tau phosphorylation in mice (Guo et al., 2013).

Similar to iron, zinc represents not only one of the most abundant metals in the body but also in the brain (Mot and Crouch, 2017). Physiologically zinc is present in free as well as in combined state, i.e., bound to proteins in the human body. Bound zinc is crucial for the function of metalloenzymes, transcription factors and signaling kinases, free zinc localizes at glutamatergic nerve terminal released upon activation, interacting with post-synaptic receptors and transporters (Mot and Crouch, 2017). Zinc imbalance leads to primary and secondary effects. Deficiency at synapse for example leads to age dependent learning and memory impairments along with increasing the overload of other biometals like iron, copper, nickel, etc. Excess zinc, however, causes oxidative stress mediated through mitochondrial dysfunction. Clear consensus on the levels of zinc in the AD brain has been a challenge, however, its role in APP processing and Aβ aggregation is certain. Secretases involved in APP cleavage are modulated by zinc since it promotes activity of ADAM10 and inhibits that of γ-secretase complex (Lammich et al., 1999). Zinc binds with Aβ with higher affinity across wider pH, than iron and copper (Yoshiike et al., 2001). Binding with zinc, renders Aβ non-degradable by masking its proteolytic cleavage site for metalloproteases (Bush et al., 1994) along with decreasing zinc bioavailability in synaptic cleft manifesting cognitive and memory impairment in AD (Deshpande et al., 2009). Further, pathological concentrations of zinc have been associated in inducing fibrillation and aggregation of tau. It cannot only directly bind with the tau monomer but also modulate its phosphorylation via kinases (GSK3β, ERK1/2, cJNK) (An et al., 2005; Pei et al., 2006).

Copper is an essential metal with integrated cellular function playing critical physiological roles as enzyme component such as in copper/zinc-superoxide dismutase (SOD1), Cytochrome c oxidase (COX) and ceruloplasmin (Cp). Thus, copper imbalance convincingly causes oxidative stress, mitochondrial dysfunction, and intracellular iron accumulation which are all established hallmarks of AD (Mot and Crouch, 2017). Role of copper in AD is not as simple and is rather complex owing to heterogeneous levels of the biometal reported in AD brain, CSF, and plasma (Li Y. et al., 2017). However, it has been established that copper interacts with and precipitates the pathological outgrowth of both Aβ and tau. Copper increases Aβ formation through GSK3β mediated phosphorylation of endogenous APP at Thr-668 (Acevedo et al., 2014). Copper binds with Aβ with high affinity to facilitate oligomer formation to eventually induce neurotoxicity via oxidative stress (Mathys and White, 2017). Thus, copper is suggested to precipitate with amyloid plaque leaving other regions of the brain deficient explaining the heterogeneous distribution in AD patient. Similar to iron and zinc, copper activates CDK5 and GSK3β pathway to promote tau phosphorylation in AD (Crouch et al., 2009). It is interesting to note that Menkes and Wilson’s disease with mutations of gene coding ATPase copper-transporting alpha and beta polypeptides (ATP7a, ATP7b) respectively, i.e., copper deregulation features neurodegeneration (Bull et al., 1993; Vulpe et al., 1993). Genetic analysis of AD patients revealed single nucleotide polymorphism in ATP7b, supporting the theory that copper dyshomeostasis exacerbate neurodegeneration (Li Y. et al., 2017).

## Development of Multi-Functional Anti-Alzheimer Agents

As the cause for AD is multiple, the molecule to be developed as drug for its treatment must incorporates many functions in a single molecule (Zhang, 2005; Bolognesi et al., 2011). The following strategies will be considered to design molecule for AD (a) Combine two molecules with well-known activities by linker (b) Modification of existing drug to increase is specificity or blood–brain barrier permeability (c) Introduce multiple functions in single molecule (Rodríguez et al., 2012). Attempts have been made to discover disease modifying drugs for AD by decreasing Aβ levels of the brain. This can be obtained by decreasing the production of Aβ through inhibiting secretases or increasing it’s removal via the use of immunization. The secretases inhibitors were failed in clinical trial due to toxicity issues such as increased skin cancer risk and cognitive performance deterioration (Extance, 2010). The first active immunotherapy trial targeting Aβ due to meningoencephalitis observed in a number of patients was terminated (Orgogozo et al., 2003). The development of meningoencephalitis may induced by T-cell response against Aβ.

Chelating agents have been used for the removal of toxic metals such as arsenic, lead from the body (Flora, 2013). As it has been revealed from many studies that imbalance of metal ions in brain is one of the major contributing factors in the advancement of AD so metal chelating therapy could be one of the strategies employed for the treatment of the AD. The chelating agents must possess properties like having moderate binding affinity for copper, zinc and iron metals, ability to cross BBB and should not toxic (Bendova et al., 2010). Metal chelators capable to chelate both free metal ions and metal ions accumulated within Aβ plaques but not remove metal ions from metalloproteins which participate in various physiological processes vital for human life are to be developed. Coordination number and binding affinity of the Cu^+2^ and Zn^+2^ ions toward Aβ peptide and metalloproteins and found less dissociation constant value for Aβ peptide than metalloproteins have been investigated (Faller and Hureau, 2009). Two compounds deferiprone (DFP) and clioquinol (CQ) are selected as the potential lead for the development of drugs against AD. The first metal based drug Deferoxamine was clinically investigated for AD. Deferoxamine is a bacterial siderophore that strongly binds iron and aluminum and a study comprises of a single-blind trial of 48 AD patients showed that the rate of reduction of cognitive decline was over 24-month period (Ayton et al., 2015b).

**FIGURE 1:**
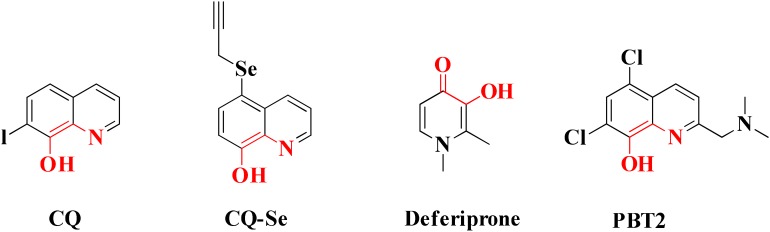
Various types of chelating agents. Red color shows metal chelating-moiety.

Common chelators such as diethylenetriamine penta acetic acid, ethylenediamine tetra acetic acid, ethylene glycol tetra acetic acid, *N,N,N,N*-Tetrakis (2-pyridylmethyl)ethylenediamine), 1,2-*bis*(*o*-aminophenoxy)ethane-*N,N,N,N*-tetra acetic acid, bathophenanthroline, and bathocuproine dissolved Aβ deposits in post-mortem brains (Atwood et al., 2004). 5-Chloro-7-iodo-8-hydroxyquinoline (clioquinol, **CQ**) was tested as metal chelating agent in AD which showed a moderate capability to bind with two metals in a square-planar geometry, ability to remove metal ions from Aβ species, capable to disaggregate Aβ aggregates but unable to stop the complete progression of Aβ aggregation (Di Vaira et al., 2004; Mancino et al., 2009; Budimir et al., 2011). Another CQ derivative **PBT2** prevented the formation **of** Aβ oligomer *in vivo* and it also dissolved the existing Aβ oligomer, enhancing cognitive function (Adlard et al., 2008; Wang Z. et al., 2014). **PBT2** in phase II clinical trial showed safety and tolerability in patients with mild AD but unfortunately the phase II imagine trial result showed no statistically significant reduction of the Aβ concentration in mild AD patients (Lannfelt et al., 2008; Sampson and Sharkey, 2008). *In vitro* experimental study of selenium containing clioquinol (**CQ-Se**) derivatives showed inhibition of metal induced Aβ aggregation, antioxidative properties and prevention of copper redox cycle. The derivative was found more active than clioquinol in radical scavenging ability, significantly inhibited Cu^+2^ induced Aβ_1–42_ aggregates and also capable to disassemble preformed Cu^+2^ induced Aβ aggregates (Wang Z. et al., 2014). Compound-(E)-*N*1,*N*1-Dimethyl-*N*4-(pyridin-2-ylmethylene)benzene-1,4-diamine has been shown to modulate reactive oxygen species (ROS) production, metal-induced Aβ aggregation, and neurotoxicity *in vitro* (Hindo et al., 2009). The drug Deferiprone used in treatment of thalassaemia can be taken as lead compound for the development of MTDL for treating AD because it has moderate to strong affinity for iron, copper and zinc, and low molecular weight makes it easily to modified into multi-functional active molecule (Rodríguez-Rodríguez et al., 2011). The study suggested that Aβ aggregation is inverted in the occurrence of Cu^+2^ and Zn^+2^ chelators. Therefore, metal chelators could be developed as therapeutic agents for the AD treatment. Figure 1 shows various types of chelating agent.

## Chelation With β-Amyloid Aggregation Inhibition

High resolution NMR spectroscopy has been used for evaluating the function of small molecules like *N*-(pyridin-2-ylmethyl)aniline (**MC-Aβ 1**) and *N*1,*N*1-dimethyl-*N*4-(pyridin-2-ylmethyl)benzene-1,4-diamine (**MC-Aβ 2**). These molecules are capable of integrating with metal ions (Zn^+2^ and Cu^+2^) and Aβ species thus act as bifunctional anti AD agents. *In vitro* studies using human neuroblastoma cell suggest that compound **MC-Aβ 2** may modulate neurotoxicity and metal-induced Aβ aggregation. Moreover, it was also found that **MC-Aβ 2** can separate Aβ aggregates of AD brain tissue homogenates containing Aβ species and metal ions (Choi et al., 2010; Braymer et al., 2011). Jones colleagues, developed many triazole based multi-functional ligands such as pyridine–triazole, quinoline–triazole, and phenol–triazole. The pyridine–triazole frameworks were synthesized by click chemistry and demonstrated to interact with the Aβ peptide as shown by NMR and docking study. These derivatives modulated Cu^+2^ and Zn^+2^ induced Aβ aggregations *in vitro* by 50–60% at pH 6.6 and 7.4 respectively as shown by turbidity assay and transmission electron microscopy. Further, ultra-violet spectroscopy was employed for testing Zn^+2^ and Cu^+2^ ions chelation ability of the pyridine-triazole derivatives (**MC-Aβ 3**). The complex was formed between metal and pyridine-triazole as revealed by X-ray crystallography study. The ligand acts as bidentate donor including nitrogen atom of pyridine and one nitrogen of triazole ring which binds to Cu^+2^ ions (Jones et al., 2012).

The quinoline–triazole (**MC-Aβ 4**) analogs modulate Aβ peptide aggregation process in the presence and absence of Cu^+2^ ions by interacting with many amino acid residues of the hydrophobic region and glutamic acid of Aβ peptide as determined by molecular modeling and 2-D ^1^H-^15^N band-selective optimized flip angle short transient heteronuclear multiple quantum correlation NMR spectroscopy. Besides these techniques, other methods such as native gel electrophoresis with western blotting and transmission electron spectroscopy were used to study ligands ability to alter Aβ aggregation. The experimental result suggested that ligands alone may not alter Aβ peptide aggregation in 24 h indicating a weak interaction between peptide and ligand but in the presence of Cu^+2^, ligands were capable to modify Aβ peptide aggregation. Therefore bidentate quinoline–triazole ligands could modulate Aβ_1–42_ peptide aggregation process in presence of excess Cu^+2^ (Jones et al., 2016). Another triazole based series of compounds were synthesized and screened for their antioxidant ability, interaction with the Aβ peptide, copper binding affinity, alteration of Aβ peptide aggregation and ability to reduce Aβ_1–42_ induced neurotoxicity. The result of the study suggests that the derivative is capable to provide protection against Aβ_1–42_ induced toxicity in human neuronal culture (**MC-Aβ 5**). The different antioxidant assays such as coumarin carboxylic acid (CCA), trolox equivalent antioxidant capacity assay (TEAC), and ascorbate reduction assay were used for the determination of antioxidant potential of the phenol–triazole derivatives. All derivatives showed TEAC values comparable to standard trolox and little bit increased values compared to CQ derivative PBT2. The CCA assay concluded that phenol–triazole compounds are not capable of binding copper and capture hydroxyl radicals. The last antioxidant assay resulted that ligands capture ROS generated by copper (Jones et al., 2017).

**FIGURE 2:**
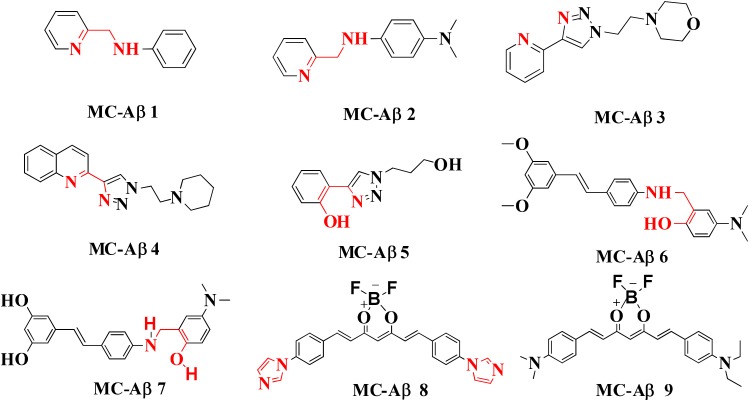
Metal chelator with Aβ aggregation inhibition. Red color shows the metal-chelating moiety.

Several experiments have suggested that a natural compound ‘resveratrol’ containing stilbene structure, act as an anti-AD agent with the capability to reduce Aβ aggregation by scavenging ROS and showing anti-inflammatory function thus effective in prevention of neurodegenerative diseases (Ono et al., 2003; Baur et al., 2006; Abraham and Johnson, 2009; Oomen et al., 2009). *In vitro* studies demonstrated that new derivatives obtained by combination of resveratrol and the structural portion responsible for metal chelation of the CQ, had significant effect on Aβ aggregation inhibition induced by self- and Cu^+2^, metal chelating properties and potential antioxidants properties. Acute toxicity and *in vitro* BBB penetration studied of the compounds **MC-Aβ 6** and **MC-Aβ 7** resulted with the positive outcome that these did not show toxicity in mice at 2000 mg/kg of doses and had BBB permeability. The IC_50_ values for **MC-Aβ 6** and **MC-Aβ 7** compounds against self-induced Aβ aggregation are 7.56 and 6.51 μM, respectively. Oxygen radical absorbance capacity using fluorescein for **MC-Aβ 6** and **MC-Aβ 7** are 4.72 and 4.70, respectively. However, there is a side effect of reported chelators that they can disturb the brain metal homeostasis after a prolonged treatment (White et al., 2006; Schäfer et al., 2007; Lu et al., 2013). Curcumin-based near-infrared fluorescence imaging probes (**MC- Aβ 8** and **MC- Aβ 9**) were designed and synthesized for detecting both soluble and insoluble Aβ species. The covalent cross linking of Aβ peptide is induced by copper via its coordination with imidazoles of histidine 13 and 14 of Aβ. The probes could attenuate Aβ cross-linking induced by copper. *In vitro* study suggested that probes changed their fluorescent characteristics when mixed with soluble and insoluble Aβ species. The fluorescence imaging probes detected the presence of soluble Aβ form in *in vivo* experiment (Zhang et al., 2013). Antiamyloidogenic property of aminoquinoline derivatives in both the absence and presence of metal ions was investigated, revealed that dimethyl amino group is the structural element essential for giving antiamyloidogenic activity (Derrick et al., 2016). Ferulic acid is a small phenolic molecule possessing strong antioxidant properties and inhibiting Aβ aggregation (Bramanti et al., 2013). Figure 2 showed metal-chelators with inhibition of Aβ aggregation.

## Chelation With Acetyl Cholinesterase Inhibition

**FIGURE 3:**
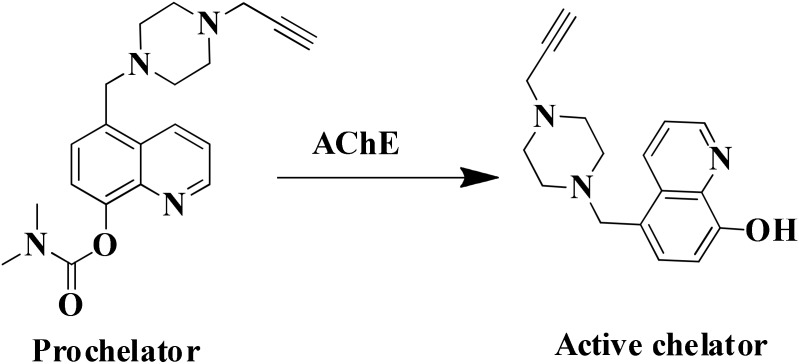
Mechanism of activation of prochelator.

Tacrine, first drug approved by FDA in 1993 acts as cholinesterase inhibitor, was removed from the drug list due to severe hepatotoxicity (Nordberg and Svensson, 1998). However, due to low molecular weight of tacrine and its effectiveness as AChE inhibitors, is used to develop hybrid or multi-target ligands (Musial et al., 2007; Chen X. et al., 2012; Ismaili et al., 2017). The tacrine based hybrids are developed as MTDL and evaluated for hepatoprotective property also for examples tacrine-NO hybrids developed as AChE inhibitor and vasorelaxation. In the series of compounds tested, one of the hydrid was not found hepatotoxic compared to drug tacrine (Fang et al., 2008a). The modification in tacrine was made at amino group linked via different type of linker to other moiety supposed to provide pharmacological activity like tacrine–melatonin, tacrine–ferulic acid, tacrine–chromene, tacrine–rhein, etc. The drug tacrine is hepatotoxic, therefore the prepared tacrine based compound mostly tested for hepatotoxicity (Rodríguez-Franco et al., 2006; Fang et al., 2008b; Pi et al., 2012; Li et al., 2014; Digiacomo et al., 2015). The derivatives of 8-hydroxyquinoline could form complex with metal ions resulted in decrease in level of β-amyloid (Aβ). A novel tacrine-8-hydroxyquinoline hybrid (**MC-AChE1**) as a potential multi-functional molecule has been synthesized and evaluated for the treatment of AD. The hybrid molecule was found more potent than tacrine in inhibiting human AChE and BuChE at nano and subnanomolar concentration, respectively. The tacrine hybrids also provided protection against free radicals and selectively chelated Cu^+2^ ions. The *in vitro* study suggested that compounds may cross BBB which is the most important property required for drugs to be approved for central nervous system disorders (Fernández-Bachiller et al., 2010). Many different hybrids of tacrine and ferulic acid are reported as potent cholinesterase inhibitors (Fang et al., 2008b; Chen Y. et al., 2012; Pi et al., 2012; Digiacomo et al., 2015). A novel tacrine–ferulic hybrid (**MC-AChE 2**) was *in vitro* evaluated against AChE and BuChE, reducing self-induced β-amyloid (Aβ) aggregation and chelating Cu^+2^. In this hybrid, the tacrine and ferulic acid linked by piperazine moiety which has the ability to bind in the mid-gorge site of AChE and it also chelated divalent Cu^+2^ ion (Xie et al., 2013; Fu et al., 2016). Zheng et al. (2009) developed a novel prochelator by incorporating the structural features of drug rivastigmine, donepezil, and **MC-MAO 2.** The prochelator is activated after binding with AChE and released an active chelator, capable of modulating the regulation of APP, reducing oxidative stress, Aβ peptide and also chelate Fe^+2^, Cu^+2^, and Zn^+2^ metal ions in the brain (Figure 3) (Zheng et al., 2009). The metal chelating ability was introduced in *bis*(7)-tacrine (**MC-AChE 3**), the resulting molecules is capable to binds with both peripheral anionic site and catalytic active site of the AChE as well as chelate metals. *In vitro* evaluation of *bis*(7)-tacrine showed activity against human AChE, inhibit the AChE-induced Aβ aggregation, and chelate iron and copper metals (Bolognesi et al., 2007). A series of Indanone derivative consists of different amine groups linked to indanone with different length of carbon spacer were synthesized and evaluated for AChE inhibition. The indanone derivative with piperidine group linked with two carbon spacer were found to inhibit AChE with IC_50_ of 0.0018 μM along with copper, zinc, and iron metal chelation ability. The Indanone derivative was found to be 14 times more potent than drug Donepezil in inhibiting AChE (Meng et al., 2012).

## Chelation With Monoamine Oxidase Inhibition

Molecules containing propargylamine group like Ladostigil, Selegiline, and Rasagiline were found neuroprotective in both *in vitro* and *in vivo* studies. Studies have reported that iron accumulation may lead to the oxidative stress and ultimately apoptosis. The level of glia hydrogen peroxide may lead to an increased MAO activity, resulting in the production of reactive hydroxyl radical. Thus the molecules containing triple bond and a portion accountable for metal chelation were developed. This strategy was applied to design hybrid molecules containing 8-hydroxy quinoline and propargyl moieties of the anti-Parkinson drug, rasagiline and selegiline (**MC MAO 1 and MC MAO 2**) (Youdim et al., 2005). Selegiline inspired compounds (**MC MAO 3**) were also designed, synthesized, and evaluated *in vitro* for MAO-B activity (IC_50_ = 0.21 μM for most potent compound), antioxidant activity (ORAC = 4.20 for most potent compound), and biometal chelating ability against Cu^+2^, Zn^+2^, Fe^+2^ (Xie et al., 2015). Further, molecular docking study was performed to evaluate binding of selegiline derivatives with MAO B. The interactions between MAO B and the compound involved face to face cation pie stacking, hydrogen bonding and hydrophobic interactions. A series of 3,5-diaryl-4,5-dihydroisoxazoles was also reported as selective MAO B inhibitors at nanomolar concentration range. The derivatives were evaluated for their ability to coordinate Fe^+2^ and Fe^+3^ cations. The compounds were found active inhibitors of MAO B but were unable to bind iron metal (Meleddu et al., 2017).

## Chelation With Antioxidant Property

Numerous studies reported the design and synthesis of hybrid molecules could chelate metals endowed with antioxidant properties (Flora et al., 2013). Phenol-diamide compound (MC-AO) was tested against AD. The properties showed by the compound include water-soluble, non-cytotoxic, capable of trapping ROS species and chelating Cu^+2^ and Fe^+3^ ions (Eckshtain-Levi et al., 2016). Analogs of adenine/guanine 2′,3′ or 3′,5′-*bis*(thio)phosphate have demonstrated promising antioxidant activity and chelation affinity to Zn^+2^ ions (Hevroni et al., 2016). Quinolone inspired molecules with a metal-binding component and antioxidant activity were designed by many research groups. Compounds exhibited antioxidant potential through binding metal ions, quenching radical ions via phenolic group thus preventing redox cycling (Braymer et al., 2011; Savelieff et al., 2014; Derrick et al., 2016). The prevention of generation of free radicals is important since it leads to oxidative damage which ultimately causes neuronal cells death.

## Chelation With Bace 1 Inhibition

**FIGURE 4:**
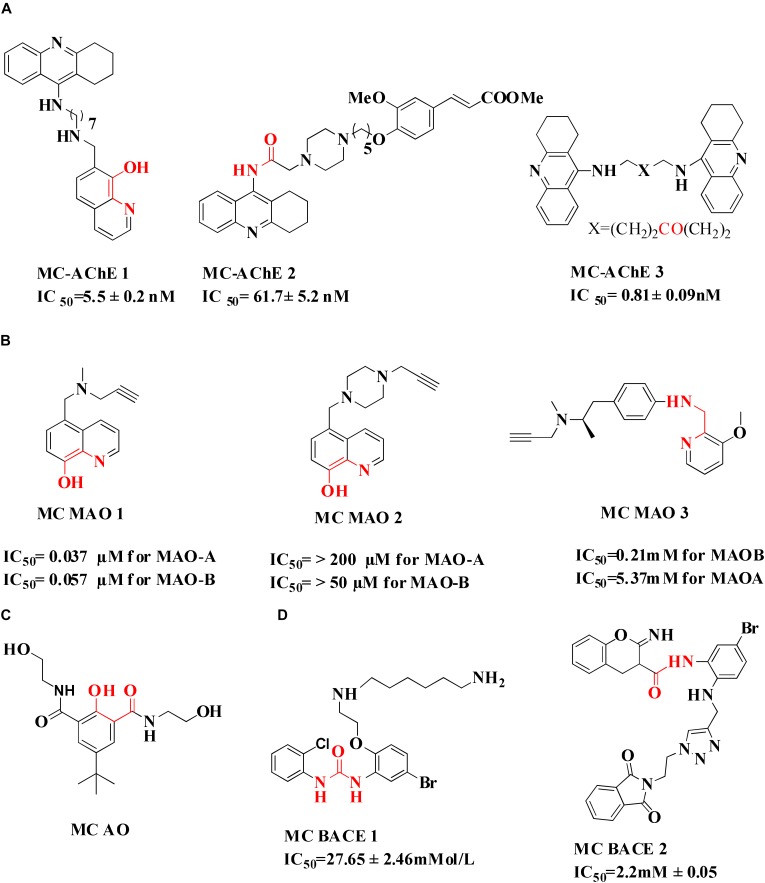
**(A)** Metal chelator with AChE inhibition; **(B)** metal chelator with MAO inhibition; **(C)** metal chelator with antioxidant; **(D)** metal chelator with BACE1 inhibitions. Red color indicates binding sites for metal chelation.

β-Secretase (memapsin 2, BACE 1) and γ-secretase are the two transmembrane aspartic protease acting on APP to generate Aβ (Selkoe and Hardy, 2016). Peptide and pseudopeptidic modified molecules such as hydroxyethylene, hydroxyethylenamine, carbylamines, acylguanidine, aminoimidazole, and aminoquinazoline are reported as BACE1 inhibitors (Ghosh et al., 2012). Huang et al. (2010) designed a series of novel 1,3-diphenylurea derivatives (**MC BACE1**) incorporating the structural features accountable to chelate metal ions with BACE 1 inhibiting potential. 1,3-Diphenylurea derivatives were investigated for their ability to chelate Cu^+2^ and Fe^+3^ ions via spectroscopic method along with BACE 1 inhibition via fluorescence resonance energy transfer assay. Docking study validated inhibition of BACE 1 by the 1,3-diphenylurea derivative. The hydrogen bonding interaction was found between Asp-228 and Thr-72 amino acids of BACE 1 and urea group of compound. The π–π stacking interaction was found between benzene ring of Tyr-71 of receptor S_1_ pocket and bromo substituted benzene portion of the compound (Huang et al., 2010). Iminochromene carboxamides containing aminomethylene triazole (**MC BACE2)** derivatives were synthesized by click chemistry and found potent inhibitor of BACE 1. The various aryl substituted groups were bonded to triazole ring and biological evaluation showed that introduction of phthalimide moiety could enhance multi-functional properties. Further molecular modeling study revealed the role of amine and amide linker which involved in hydrogen bonding with amino acids of the active site of BACE 1. *In vitro* neuroprotection study on PC12 neuronal cells was also found promising, suggested that these derivatives could be taken as multi-functional lead compound for further study (Iraji et al., 2017). Figure 4 shows molecules. Folk colleagues, reported a prochelator that is activated by enzyme BACE 1 to release an active molecule which has affinity to chelate Cu^+2^ ions. The activated molecule is capable to sequesters Cu^+2^ from Aβ thus preventing and disaggregating Aβ along with a capacity to reduce copper promoted ROS formation (Folk and Franz, 2010).

## Molecules With Multi-Functional Pharmacological Properties

**FIGURE 5:**
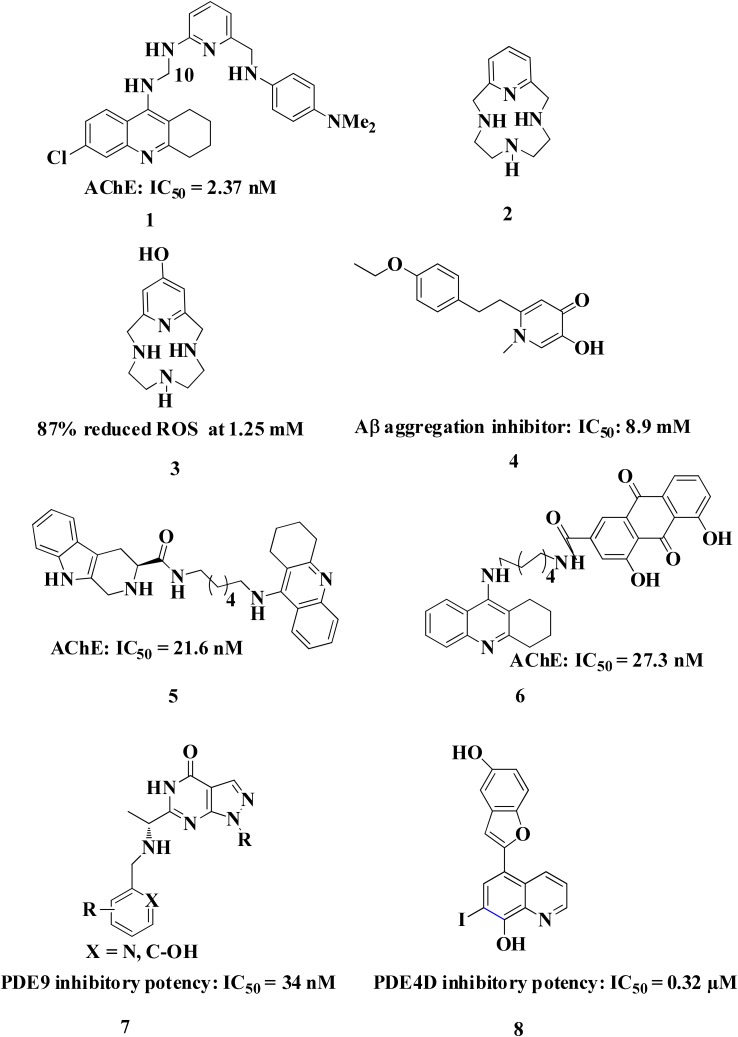
Structures of MTDL.

In the earlier sections we described some molecules capable of chelating metals along with one more factor involved in AD development. This section includes molecules targeting more than one factor along with metal chelation such as a hybrid of 6-chlorotacrine and a metal-Aβ modulator that inhibits AChE, metal-free/metal-induced Aβ aggregation and chelate metals (Kochi et al., 2013; Lee et al., 2013). These hybrids were evaluated for their potential against AChE (IC_50_ = 2.37 nM for most potent hybrid) (Figure 5:1), metal-free and metal-induced Aβ_1–42_ aggregation inhibition, disaggregation of the same and interaction with biometal ions such as Cu^+2^ and Zn^+2^ (Kochi et al., 2013). Pyclen (tetraazamacrocycles; Figure 5:2) and their derivatives (Figure 5:3) are also reported as antioxidant, ability to inhibit metal promoted Aβ formation, metal ion chelation and capability to provide protection against ROS induced cell death (Green et al., 2010; Lincoln et al., 2012, 2013). Resveratrol, a natural antioxidant agent and β-amyloid peptide (Aβ) aggregation inhibitor were used in the designing of many multi-functional ligands for example, fusing it with some other molecule possessing other biological activity such as metal chelation (Table 2). *In vitro* biological evaluation of series deferiprone–resveratrol hybrids (Figure 5:4) exhibited excellent antioxidant activity, good inhibitory activity against self-induced Aβ_1–42_ aggregation in micro molar range, and a potent metal chelating capacity (Xu et al., 2017). Another novel series of compounds were synthesized by combining the pharmacophores of resveratrol and clioquinol, showed excellent MTDL properties like possessing significant capability to inhibit Cu^+2^ induced Aβ aggregation and self-induced β-amyloid (Aβ) aggregation, potential antioxidant behavior and biometal chelation (Mao et al., 2014). *In vitro* studies of schiff base of 4-hydroxycoumarin showed their abilities to inhibit MAO, self-induced and Cu^2+^-induced β-amyloid aggregation, as well as acting as potential biometal chelators and antioxidants therefore these could be a promising molecules act as MTDLs (Wang Z.-M. et al., 2015). Phenolic mannich base bearing molecules display AChE inhibitory activity, antioxidant, and metal chelating properties as reported in several studies (Park et al., 2012; Büyükkidan and Özer, 2013; Roman, 2015). Hybrids of pyridoxine and resveratrol with mannich base moieties were synthesized and found that they could selectively inhibit AChE, MAO-B, exhibited antioxidant and metal chelating properties (Yang et al., 2017). 4′,5,6-Trihydroxyflavone-7-glucuronide (scutellarin) is a major active component in breviscapine isolated from Chinese herb *Erigeron breviscapus* (vant.) Hand. Mazz exhibits various pharmacological activities especially for neurological disorder. Sang et al. (2015) designed a hybrid molecule based on scutellarin and rivastigmine. They have incorporated carbamate moiety of rivastigmine in the core structure of the scutellarin.

**Table 2:** Multi-functional molecules as anti-Alzheimer’s agent (Red color indicates metal-chelating portion).

#	Molecules	Activity	Metal chelator	Reference
1	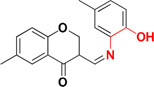	MAO-A: IC_50_ = 5.12 μM MAO-B: IC_50_ = 0.816 μM Aβ aggregation inhibition: 75.1% at 20 μM Antioxidant activity: ORAC = 3.62 of trolox equivalent Reduce PC12 cells death induced by oxidative stress. Penetrate the BBB	Fe^+2^, and Fe^+3^ Cu^+2^, Zn^+2^	Li F. et al., 2017

2	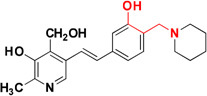	AChE : IC_50_ = 2.11 μM BuChE: 16.7% MAO-A: IC_50_ = 12.8 μM MAO-B: IC_50_ = 12.4 μM Antioxidant activity: ORAC: 2.56 of trolox equivalent	Cu^+2^, Zn^+2^, and Fe^+2^	Yang et al., 2017

3	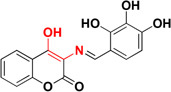	MAO-A: IC_50_ = 0.673 μM MAO-B: IC_50_ = 0.711 μM Antioxidant activity: ABTS: 1.34 of trolox equivalent. DPPH: IC_50_ = 45.8 μM Self-induced Aβ_1–42_ aggregation inhibition: 60.1% at 20 μM. Cu^2+^ -induced Aβ_1–42_ aggregation inhibition of 45.7% at 50 μM showed neuroprotective effects in PC_12_ cells	Cu^+2^	Wang Z.-M. et al., 2015

4	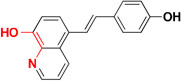	Inhibits Aβ aggregation: IC_50_ = 8.50 μM Antioxidant activity: ORAC: 2.18 μmol of Trolox equivalent	Cu^+2^	Mao et al., 2014

5	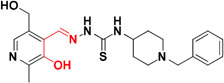	AChE: IC_50_ = 4.93 μM Inhibits Aβ aggregation oxidative stress	Fe^+2^	Palanimuthu et al., 2017

6	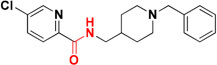	AChE Inhibition: IC_50_ = 0.220 mM butyrylcholinesterase Inhibition: IC_50_ = 1.23 mM MAO-B: IC_50_ = 3.14 mM MAO-A: 13.4 mM	Cu^+2^	Li et al., 2016

7	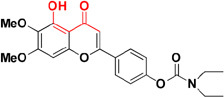	AChE Inhibition: IC_50_ = 0.57 μM Butyrylcholinesterase Inhibition: IC_50_ = 22.6 μM Antioxidative activity: ORAC: 1.3 of trolox equivalent	Cu^+2^	Sang et al., 2015

8	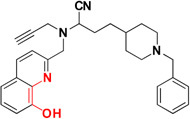	hAChE:IC_50_ = 29 nM hAChE:IC_50_ = 39 nM hMAO-A: IC_50_ = 10 mM hMAO-A:IC_50_ = 100 mM antioxidant property	Cu^+2^ and Zn^+2^	Wu et al., 2016

9	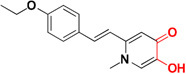	Self-induced Aβ aggregation: IC_50_ = 8.9 μM Antioxidant property: ABTS assay: IC_50_ = 4.02 ± 0.34 μM by trolox equivalent	Fe^+3^	Xu et al., 2017

10	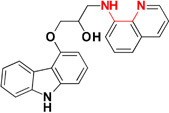	Inhibition of copper-mediated and self mediated Aβ_1–42_ aggregation Neuroprotective effect Low toxicity	Cu^+2^	Zhang et al., 2018

11	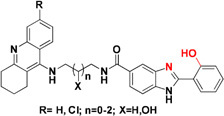	AChE inihibition:IC_50_ = 6 nM Aβ aggregation inhibition: 74.6% Neuroprotector Moderate radical scavenger	Cu^+2^ and Zn^+2^	Hiremathad et al., 2018

12	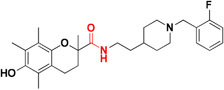	hAChE inhibition:IC_50_ = 0.54 μM hMAO-B inhibition: IC_50_ = 4.3 μM, Antioxidant activity: DPPH: 41.33 μM ABTS: IC_50_ = 1.72 by trolox equivalent ORAC: IC_50_ = 1.79 by trolox equivalent	Cu^+2^	Cai et al., 2017

The naturally occurring molecules “coumarins” and “flavonoids” exhibit wide range of pharmacological properties. Hence, researchers fascinated to take these scaffold for designing MTDL (Katalinić et al., 2010; Lou et al., 2011; Uriarte-Pueyo and Calvo, 2011; He et al., 2012). Tacrine–coumarins hybrids inhibited ChE significantly, self-induced Aβ_1–42_ aggregation and may also function as an excellent metal chelators. The kinetic and molecular modeling studies suggested that they are capable of binding catalytic active site, peripheral and mid-gorge sites of AChE. This type of interaction suggests that tacrine–coumarins hybrids function as a mixed-type inhibitor (Li et al., 2013; Xie et al., 2013). A novel series of tacrine–flavonoid hybrids were found to be potent and showed balanced inhibitory profile against self-induced Aβ_1–42_ aggregation, ChE, metal chelating property (Cu^+2^ and Fe^+2^) and low cell toxicity suggesting that it might be an excellent MTDL for the treatment of AD. The binding interaction of tacrine–flavonoid hybrids with target AChE was same as showed by tacrine–coumarins hybrids (Li et al., 2013). The extensive research on flavonoid based derivatives revealed that amine group are critical for inhibition of AChE, hydroxyl, type of substitution and linker in flavones scaffolds modulate biological activities (Jalili-Baleh et al., 2018). A series of hybrid of tacrine linked with appropriate alkylidene linker to β carboline (Figure 5:5) were reported as multi-functional anti-Alzheimer agents. The tacrine–carboline hybrids were tested for various pharmacological activities such as *in vitro* inhibition of AChE and BuChE, self and Cu^+2^ induced Aβ_1–42_ aggregations, ability to chelate Cu^+2^ ion. It showed potent inhibition of AChE with IC_50_, 21.6, 63.2 nM for hAChE and 39.8 nM for BuChE, inhibits 65.8% Aβ aggregation at 20 μM, antioxidant activity was found 1.57 trolox equivalent, reduce neuroblastoma cell lines PC12 cells death and crosses the blood–brain barrier as determined by parallel artificial membrane permeation assay (Lan et al., 2014).

Tacrine hybrids with benzoquinone or anthraquinone have been synthesized and evaluated for multi-functional activities. Among the tested compounds, one hybrid (Figure 5:6) showed *in vitro* AChE inhibition in the nanomolar range and was also five times more potent than drug tacrine itself. The same compound also showed a moderate inhibition of BuChE with IC_50_ value of 200 nM. Further, computational and kinetic data suggest that hybrid are capable of simultaneously binding to CAS and PAS of AChE thus it behaves like a mixed- type of inhibitor. Apart from this, tacrine–rhein hybrid is also capable of inhibiting AChE induced Aβ aggregation, could chelate Cu^+2^ and Fe^+2^ metals and less hepatoxic and safer than tacrine as showed by *in vivo* experiment. They measured level of aspartate aminotransferase and alanine aminotransferases in heparinised serum of adult mice at different interval of times. The activities of these enzymes were low in compound treated mice suggesting that tacrine–rhein hybrids are safer than tacrine itself in terms of hepatoxicity (Li et al., 2014).

Tacrine hybrids with hydroxyphenylbenzimidazole possess multi-functional properties like it inhibits AChE at nanomolar concentration, inhibits self as well as Cu^+2^ induced Aβ aggregation, metal chelating ability, radical scavenging activity and neuroprotection (Hiremathad et al., 2018). The tacrine and hydroxyphenylbenzimidazole hybrids linked via a carbon spacer with varying length from one to three carbon atoms and the hydroxyl group bond to carbon linker has also been used as linker. The most potent hybrid inhibits AChE with IC_50_ value of 6 nM. The hybrid linked via three carbon spacer inhibits 74% Aβ aggregation. The best antioxidant activity was showed by a hybrid linked with hydroxyl group which also chelates Cu^+2^ and Zn^+2^ ions. A series of 8-hydroxyquinoline derivatives as MTDL were also investigated. The pharmacological properties such as inhibition of self-induced and Cu^+2^/Zn^+2^ Aβ_1–42_ aggregations were determined with IC_50_ value of 5.64 μM for the former inhibition. The antioxidant ability of the most potent derivatives was found to be nearly 2.63 trolox equivalents. The molecules chelated Cu^+2^ and Zn^+2^, possess low neurotoxicity in PC12 cells and capable of penetrating BBB *in vitro.* No acute toxicity at doses up to 2000 mg/kg in mice was reported for the derivatives (Yang et al., 2018). Table 2 represents various MTDL with their biological properties.

Cyclic adenosine monophosphate and cyclic guanosine monophosphate are cyclic nucleotides involved in a number of cellular activities such as neuroplasticity, neuroprotection, and memory function. Degradation of cyclic nucleotides is catalyzed via phosphodiesterase leading to the development of phosphodiesterase inhibitors which may prolong cAMP signaling, playing a vital role in the regulation of various biochemical processes. Molecules have been designed by incorporating metal chelator such as clioquinol and moiety capable of inhibiting phosphodiesterase. A series of clioquinol-moracin hybrids (Figure 5:7) were evaluated for multi-functional activities such phosphodiesterase 4D inhibition, Aβ aggregation inhibition antioxidant effects, biometal chelating ability, blood–brain barrier permeability and neuroprotective effect against inflammation in microglial cells (Wang Z. et al., 2015). An interesting design for a novel series of compounds was suggested recently based on clioquinol and PF-04447943 (Figure 5:8) were reported as metal induced Aβ_1–42_ aggregation inhibitor, ability to prevent copper redox cycle and inhibits phosphodiesterase (Su et al., 2016).

## Beneficial Effects of Natural Product Against AD

Epidemiological and experimental evidences suggest that various foods items and their chemical constituents may have beneficial effects in various diseases and in particular metal intoxication (Flora, 2011). A balanced food can acts as a disease-modifying agent in AD. Numerous studies have shown that foods rich in curcumin, epigallocatechin gallate, gallic acid, L-ascorbic acid, and α-tocopherol consumed by older people in their diet, may have a better memory function (Ringman et al., 2005; Bastianetto et al., 2006; Ng et al., 2006, 2008; Eskelinen et al., 2009; Dysken et al., 2014; Harrison et al., 2014). Diet consists of citrus fruits, nuts, curries, tea, and coffee can also provide chemical constituents useful in AD (Dysken et al., 2014; Harrison et al., 2014).

**FIGURE 6:**
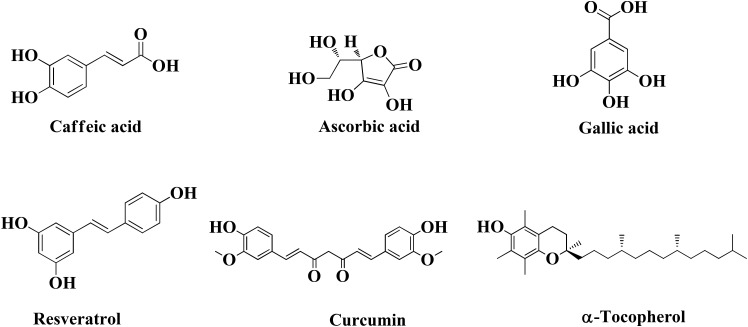
Structures of natural molecule beneficial against AD.

An interesting study conducted to investigate different food constituents such as (-)-epigallocatechin gallate (EGCG), L-ascorbic acid, caffeine, caffeic acid, curcumin, gallic acid, resveratrol, α-tocopherol, and propyl gallate provided some useful data against AD (Figure 6). These food constituents were mainly evaluated as their capability to chelate metals, antioxidant, Aβ_1–42_ fibrillation and suggested that food constituents have strong to moderate abilities to chelate metals (Cu^+2^, Zn^+2^, and Fe^+2^), inhibit Aβ_1–42_ fibrillation as well as scavenge free radicals (Chan et al., 2016). Enol form of curcumin possesses Cu^+2^ and Zn^+2^ ions chelation ability as its phenolic OH group is capable to scavenge free radical thus producing resonance stabilized phenoxy radical (Priyadarsini et al., 2003; Zhao et al., 2010). The dichloromethane leaves and roots extracts of Juncaceae species (*J. maritimus*, *J. acutus*, and *J. inflexus*) were evaluated for properties like *in vitro* cholinesterase inhibition, radical scavenging and metal chelation. The extracts were tested against various radicals like DPPH, ABTS, and FRAP and outcome of the study showed different level of trapping of radicals. Other than this dichloromethane extract of leaves from *J. acutus* strongly inhibited BuChE. The bioactive compound isolated was ‘’Juncunol” which inhibits cholinesterase in neuronal and glial cells *in vitro*, suggested that Juncus spp. can be used as nutraceutical for AD patients (Rodrigues et al., 2017). There is a correlation between the increase levels of Aβ and increase of homocysteine levels which may be attributed to the deficiency of pyridoxine. Preclinical and clinical studies specify that supplementation of vitamins like pyridoxine, folic acid, cobalamine, vitamin A, and vitamin K may be included in the treatment of AD (Bhatti et al., 2016). Therefore, food constituents can reduce Aβ fibrillate ion, chelate metal ions, and scavenge free radicals. Table 3 provides information about the molecules which are under clinical trial.

**Table 3:** Chelators and natural molecules under clinical trial (Babitha et al., 2014; Xu et al., 2015).

#	Drugs	Study undertaken	Phase
1	Deferiprone	Mild AD	Phase II
2	CQ	• Slow down the process of cognitive impairment. • Found to lower plasma Aβ levels. • CQ causes sub-acute myelooptic neuropathy on long term usage. • Due to neurotoxicity and mutagenic effect, study has been suspended.	Phase IIa
3	PBT2	Study showed no significant decrease in the Aβ levels in mild AD patients.	Phase IIa
4	Resveratrol	It has potential to inhibit Aβ aggregation.	Phase III
5	Benfotiamine	Minimize cognitive decline in mild AD patient.	Phase II
6	Huperzine A	It is used in China for the treatment of AD.	Phase III
7	ZT-1	ZT-1 is a pro-drug of Huperzine, used in China for memory disorders.	Phase II
8	7β-OH epiandrosterone	Its efficacy is tested for AD.	Phase I
9	Longvida	Curcumin formulation is evaluated for AD patient.	Phase II
10	Bryostatin-1	Treatment of AD	Phase II
11	Scyllo-inositol	Mild to moderate AD	Phase II

## Future Perspectives and Conclusion

Neurodegenerative diseases are currently a major area of concern particularly related to medical treatment. A specific and focused research toward the development of an effective drug must be made for suggesting a drug molecule which can be employed to control and provide relief to suffer population. This review is an attempt to provide the readers a critical evaluation of available data for AD, a neurodegenerative disorder and specifically suggesting chelating agents as MTDL which could be developed as drug. The findings that there is a link between abnormal protein folding and metal imbalances, contribute in pathogenesis of AD, offer researcher to design a multi-functional molecule especially combining metal chelating property in it. Multi-functional ligands can be developed by incorporating potential activity for cholinesterase inhibition, MAOs inhibition, BACE 1 inhibition, capable with Aβ interaction, metal chelation, manage to minimize generation of ROS and antioxidant activity. Although, it looks a very difficult approach but can be achieved by extensive focused research.

Alzheimer’s disease, a complex neurodegenerative disease has limited therapeutic options. The available treatment only provides symptomatic relief in the cognitive impairment while no drug is available which may cure the disease. The multi-factorial nature of the disease makes the search for new drug a difficult task. One of the strategies to tackle a complex disease like AD, MTDL has recently been suggested. The rational design of the molecules is based on incorporating structural features in a molecule which might be responsible for the targeted activity. Thus, the designed molecule should be capable of acting on multiple targets of AD. Though, single target molecules have more affinity toward target than multi-target molecules, which generally show less affinity for the targets but possess a balanced affinity for more than one target. The property is useful to handle a complex disease where more than one factors might be responsible for the disease. Studies are in progress to determine if MTDL might be the potential strategy for the management of AD. Several *in vitro* and *in vivo* studies have shown promising results but clinical trials need to be done to prove their potential/efficacy as drug. Although, MTDL strategy has both limitations and strengths but still gaining importance in our search to find novel molecules which could be effective against AD.

## Author Contributions

All authors listed have made a substantial, direct and intellectual contribution to the work, and approved it for publication.

## Conflict of Interest Statement

The authors declare that the research was conducted in the absence of any commercial or financial relationships that could be construed as a potential conflict of interest.
